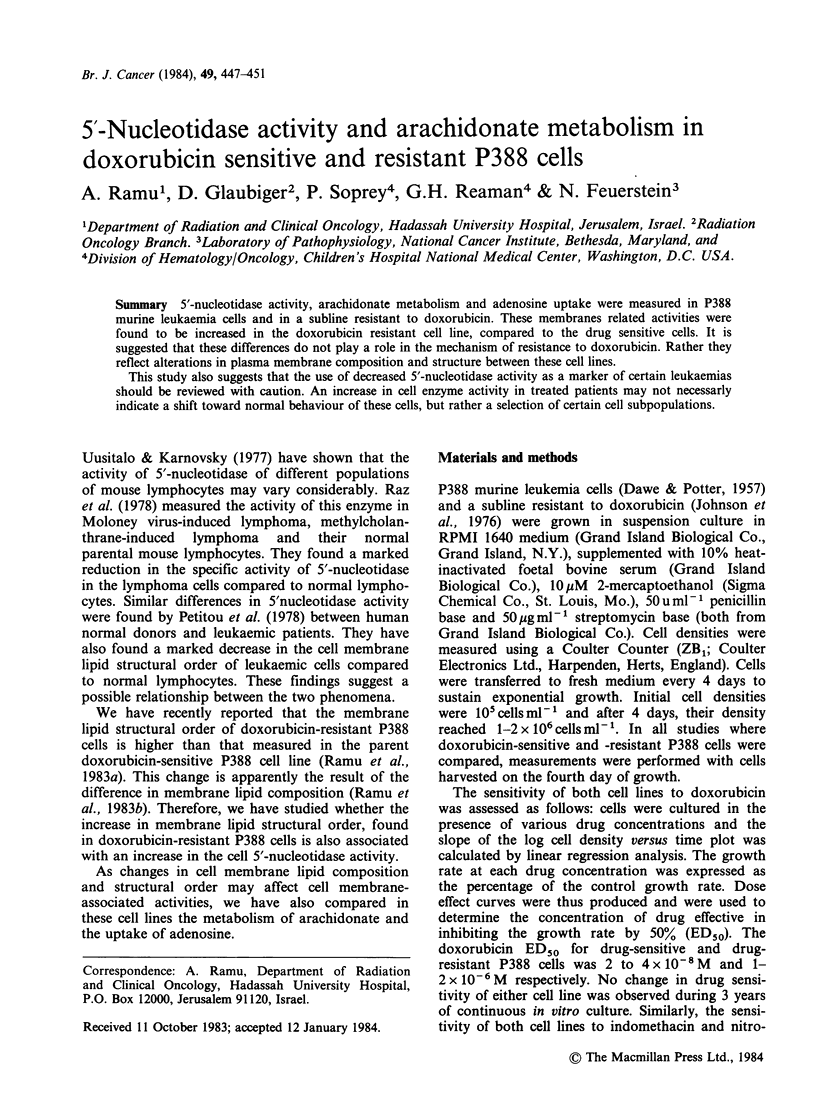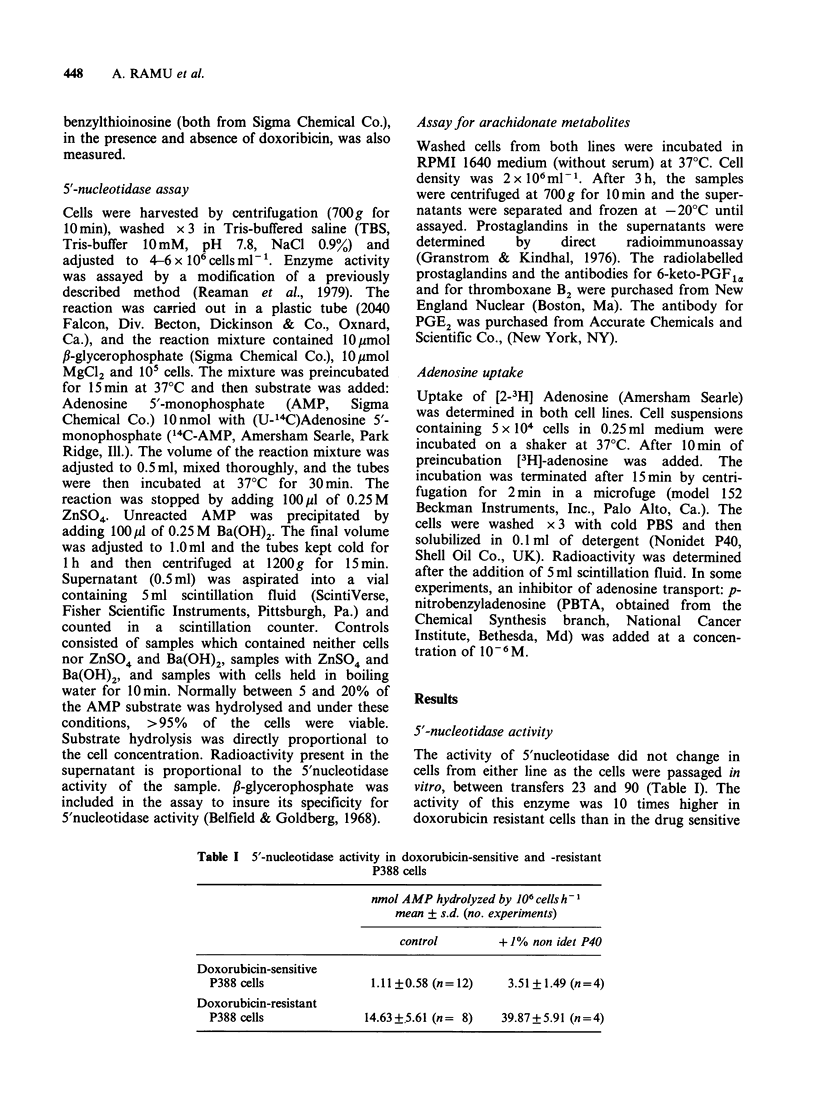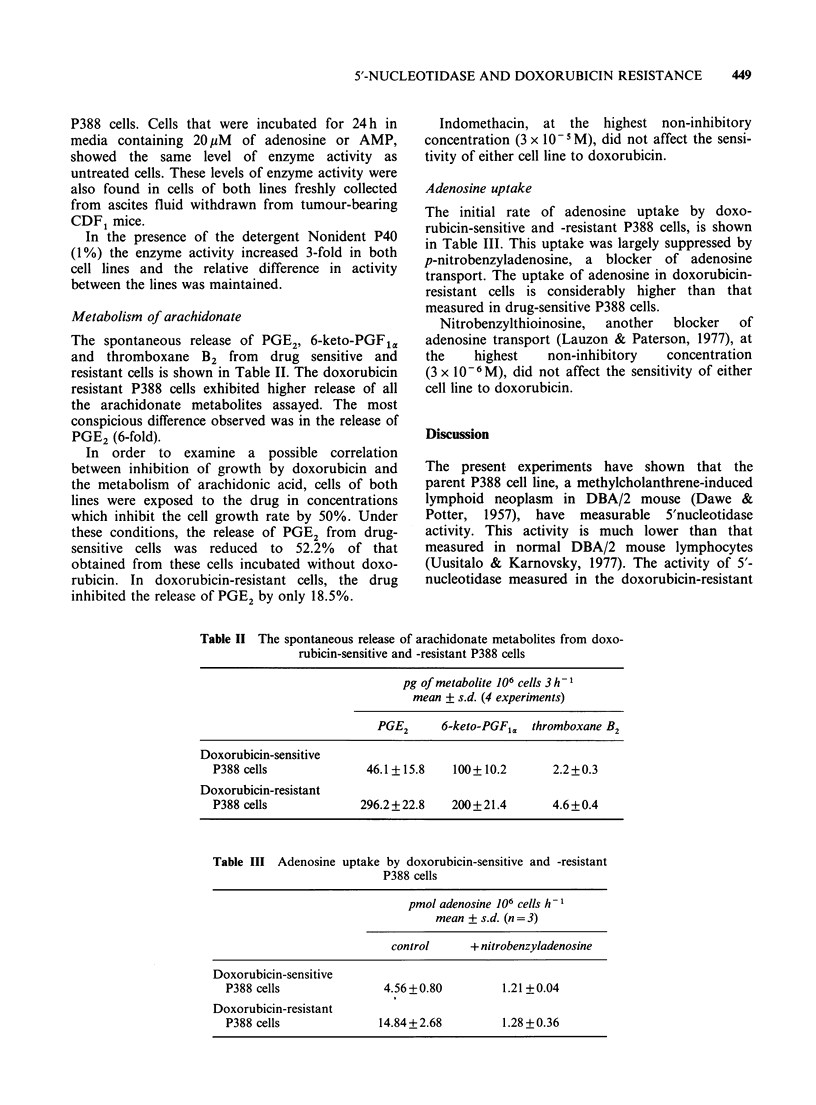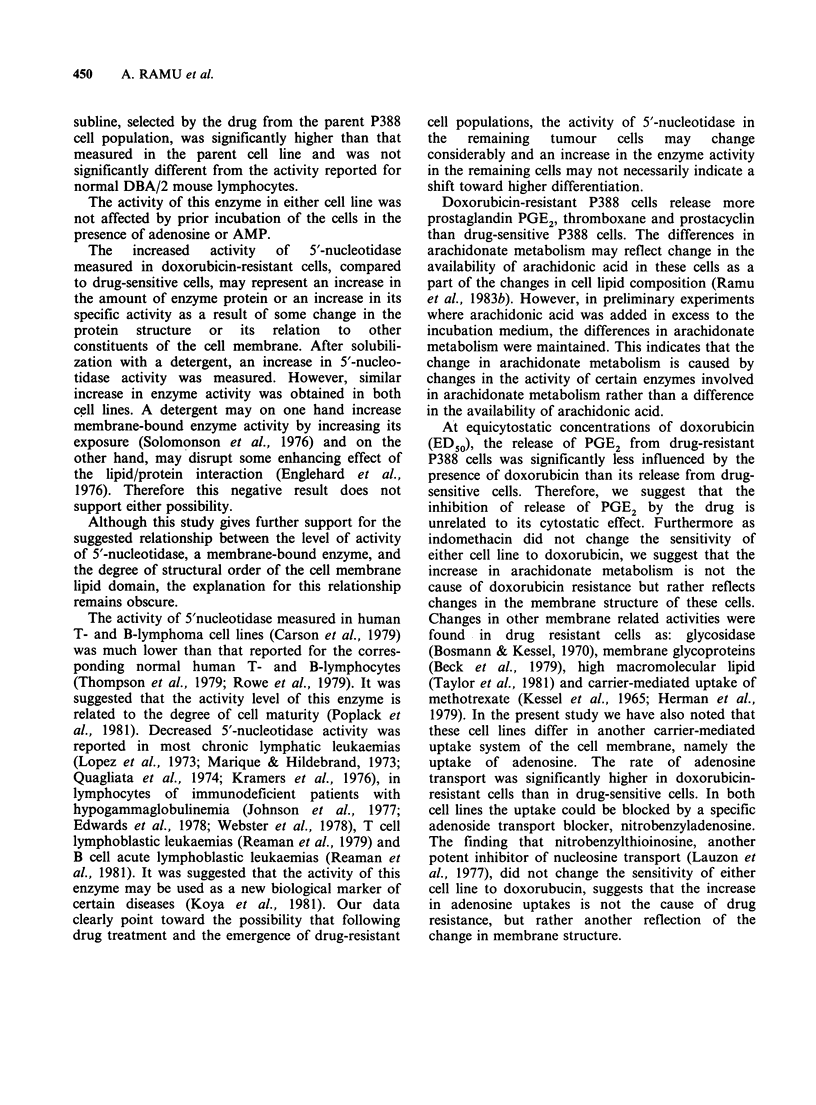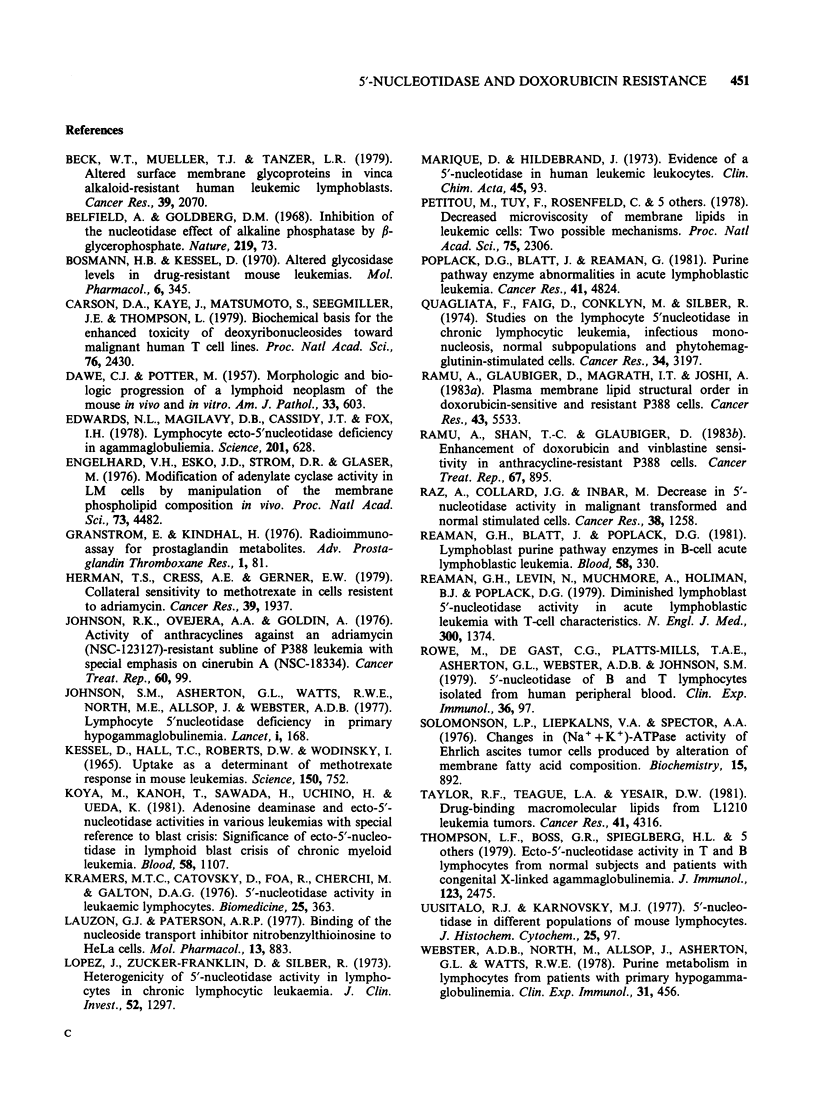# 5'-Nucleotidase activity and arachidonate metabolism in doxorubicin sensitive and resistant P388 cells.

**DOI:** 10.1038/bjc.1984.71

**Published:** 1984-04

**Authors:** A. Ramu, D. Glaubiger, P. Soprey, G. H. Reaman, N. Feuerstein

## Abstract

5'-nucleotidase activity, arachidonate metabolism and adenosine uptake were measured in P388 murine leukaemia cells and in a subline resistant to doxorubicin. These membranes related activities were found to be increased in the doxorubicin resistant cell line, compared to the drug sensitive cells. It is suggested that these differences do not play a role in the mechanism of resistance to doxorubicin. Rather they reflect alterations in plasma membrane composition and structure between these cell lines. This study also suggests that the use of decreased 5'-nucleotidase activity as a marker of certain leukaemias should be reviewed with caution. An increase in cell enzyme activity in treated patients may not necessarily indicate a shift toward normal behaviour of these cells, but rather a selection of certain cell subpopulations.


Br. J. Cancer (1984), 49, 447–451

### 5′-Nucleotidase activity and arachidonate metabolism in doxorubicin sensitive and resistant P388 cells

A. Ramu[1], D. Glaubiger[2], P. Soprey[4], G.H. Reaman[4] & N. Feuerstein[3]

[1]*Department of Radiation and Clinical Oncology, Hadassah University Hospital, Jerusalem, Israel.* [2]*Radiation Oncology Branch.* [3]*Laboratory of Pathophysiology, National Cancer Institute, Bethesda, Maryland, and* [4]*Division of Hematology/Oncology, Children's Hospital National Medical Center, Washington, D.C. USA.*

**Summary** 5′-nucleotidase activity, arachidonate metabolism and adenosine uptake were measured in P388 murine leukaemia cells and in a subline resistant to doxorubicin. These membranes related activities were found to be increased in the doxorubicin resistant cell line, compared to the drug sensitive cells. It is suggested that these differences do not play a role in the mechanism of resistance to doxorubicin. Rather they reflect alterations in plasma membrane composition and structure between these cell lines.

This study also suggests that the use of decreased 5′-nucleotidase activity as a marker of certain leukaemias should be reviewed with caution. An increase in cell enzyme activity in treated patients may not necessarly indicate a shift toward normal behaviour of these cells, but rather a selection of certain cell subpopulations.

Uusitalo & Karnovsky (1977) have shown that the activity of 5′-nucleotidase of different populations of mouse lymphocytes may vary considerably. Raz *et al.* (1978) measured the activity of this enzyme in Moloney virus-induced lymphoma, methylcholanthrane-induced lymphoma and their normal parental mouse lymphocytes. They found a marked reduction in the specific activity of 5′-nucleotidase in the lymphoma cells compared to normal lymphocytes. Similar differences in 5′nucleotidase activity were found by Petitou *et al.* (1978) between human normal donors and leukaemic patients. They have also found a marked decrease in the cell membrane lipid structural order of leukaemic cells compared to normal lymphocytes. These findings suggest a possible relationship between the two phenomena.

We have recently reported that the membrane lipid structural order of doxorubicin-resistant P388 cells is higher than that measured in the parent doxorubicin-sensitive P388 cell line (Ramu *et al.*, 1983*a*). This change is apparently the result of the difference in membrane lipid composition (Ramu *et al.*, 1983*b*). Therefore, we have studied whether the increase in membrane lipid structural order, found in doxorubicin-resistant P388 cells is also associated with an increase in the cell 5′-nucleotidase activity.

As changes in cell membrane lipid composition and structural order may affect cell membrane-associated activities, we have also compared in these cell lines the metabolism of arachidonate and the uptake of adenosine.

Correspondence: A. Ramu, Department of Radiation and Clinical Oncology, Hadassah University Hospital, P.O. Box 12000, Jerusalem 91120, Israel.

Received 11 October 1983; accepted 12 January 1984.

### Materials and methods

P388 murine leukemia cells (Dawe & Potter, 1957) and a subline resistant to doxorubicin (Johnson *et al.*, 1976) were grown in suspension culture in RPMI 1640 medium (Grand Island Biological Co., Grand Island, N.Y.), supplemented with 10% heat-inactivated foetal bovine serum (Grand Island Biological Co.), 10 $\mu$M 2-mercaptoethanol (Sigma Chemical Co., St. Louis, Mo.), 50 u ml$^{-1}$ penicillin base and 50 $\mu$g ml$^{-1}$ streptomycin base (both from Grand Island Biological Co.). Cell densities were measured using a Coulter Counter ($ZB_1$; Coulter Electronics Ltd., Harpenden, Herts, England). Cells were transferred to fresh medium every 4 days to sustain exponential growth. Initial cell densities were $10^5$ cells ml$^{-1}$ and after 4 days, their density reached 1–2 $\times 10^6$ cells ml$^{-1}$. In all studies where doxorubicin-sensitive and -resistant P388 cells were compared, measurements were performed with cells harvested on the fourth day of growth.

The sensitivity of both cell lines to doxorubicin was assessed as follows: cells were cultured in the presence of various drug concentrations and the slope of the log cell density *versus* time plot was calculated by linear regression analysis. The growth rate at each drug concentration was expressed as the percentage of the control growth rate. Dose effect curves were thus produced and were used to determine the concentration of drug effective in inhibiting the growth rate by 50% ($ED_{50}$). The doxorubicin $ED_{50}$ for drug-sensitive and drug-resistant P388 cells was 2 to $4 \times 10^{-8}$ M and 1–$2 \times 10^{-6}$ M respectively. No change in drug sensitivity of either cell line was observed during 3 years of continuous *in vitro* culture. Similarly, the sensitivity of both cell lines to indomethacin and nitro-

© The Macmillan Press Ltd., 1984

benzylthioinosine (both from Sigma Chemical Co.), in the presence and absence of doxoribicin, was also measured.

*5′-nucleotidase assay*

Cells were harvested by centrifugation (700*g* for 10 min), washed ×3 in Tris-buffered saline (TBS, Tris-buffer 10 mM, pH 7.8, NaCl 0.9%) and adjusted to 4–6×$10^6$ cells $ml^{-1}$. Enzyme activity was assayed by a modification of a previously described method (Reaman *et al.*, 1979). The reaction was carried out in a plastic tube (2040 Falcon, Div. Becton, Dickinson & Co., Oxnard, Ca.), and the reaction mixture contained 10 μmol β-glycerophosphate (Sigma Chemical Co.), 10 μmol $MgCl_2$ and $10^5$ cells. The mixture was preincubated for 15 min at 37°C and then substrate was added: Adenosine 5′-monophosphate (AMP, Sigma Chemical Co.) 10 nmol with (U-$^{14}$C)Adenosine 5′-monophosphate ($^{14}$C-AMP, Amersham Searle, Park Ridge, Ill.). The volume of the reaction mixture was adjusted to 0.5 ml, mixed thoroughly, and the tubes were then incubated at 37°C for 30 min. The reaction was stopped by adding 100 μl of 0.25 M $ZnSO_4$. Unreacted AMP was precipitated by adding 100 μl of 0.25 M $Ba(OH)_2$. The final volume was adjusted to 1.0 ml and the tubes kept cold for 1 h and then centrifuged at 1200*g* for 15 min. Supernatant (0.5 ml) was aspirated into a vial containing 5 ml scintillation fluid (ScintiVerse, Fisher Scientific Instruments, Pittsburgh, Pa.) and counted in a scintillation counter. Controls consisted of samples which contained neither cells nor $ZnSO_4$ and $Ba(OH)_2$, samples with $ZnSO_4$ and $Ba(OH)_2$, and samples with cells held in boiling water for 10 min. Normally between 5 and 20% of the AMP substrate was hydrolysed and under these conditions, >95% of the cells were viable. Substrate hydrolysis was directly proportional to the cell concentration. Radioactivity present in the supernatant is proportional to the 5′nucleotidase activity of the sample. β-glycerophosphate was included in the assay to insure its specificity for 5′nucleotidase activity (Belfield & Goldberg, 1968).

*Assay for arachidonate metabolites*

Washed cells from both lines were incubated in RPMI 1640 medium (without serum) at 37°C. Cell density was 2×$10^6$ $ml^{-1}$. After 3 h, the samples were centrifuged at 700*g* for 10 min and the supernatants were separated and frozen at −20°C until assayed. Prostaglandins in the supernatants were determined by direct radioimmunoassay (Granstrom & Kindhal, 1976). The radiolabelled prostaglandins and the antibodies for 6-keto-$PGF_{1\alpha}$ and for thromboxane $B_2$ were purchased from New England Nuclear (Boston, Ma). The antibody for $PGE_2$ was purchased from Accurate Chemicals and Scientific Co., (New York, NY).

*Adenosine uptake*

Uptake of [2-$^3$H] Adenosine (Amersham Searle) was determined in both cell lines. Cell suspensions containing 5×$10^4$ cells in 0.25 ml medium were incubated on a shaker at 37°C. After 10 min of preincubation [$^3$H]-adenosine was added. The incubation was terminated after 15 min by centrifugation for 2 min in a microfuge (model 152 Beckman Instruments, Inc., Palo Alto, Ca.). The cells were washed ×3 with cold PBS and then solubilized in 0.1 ml of detergent (Nonidet P40, Shell Oil Co., UK). Radioactivity was determined after the addition of 5 ml scintillation fluid. In some experiments, an inhibitor of adenosine transport: *p*-nitrobenzyladenosine (PBTA, obtained from the Chemical Synthesis branch, National Cancer Institute, Bethesda, Md) was added at a concentration of $10^{-6}$ M.

### Results

*5′-nucleotidase activity*

The activity of 5′nucleotidase did not change in cells from either line as the cells were passaged *in vitro*, between transfers 23 and 90 (Table I). The activity of this enzyme was 10 times higher in doxorubicin resistant cells than in the drug sensitive

**Table I** 5′-nucleotidase activity in doxorubicin-sensitive and -resistant P388 cells

| | *nmol AMP hydrolyzed by $10^6$ cells $h^{-1}$ mean ± s.d. (no. experiments)* | |
|---|---|---|
| | *control* | *+1% non idet P40* |
| Doxorubicin-sensitive P388 cells | 1.11±0.58 (*n*=12) | 3.51±1.49 (*n*=4) |
| Doxorubicin-resistant P388 cells | 14.63±5.61 (*n*= 8) | 39.87±5.91 (*n*=4) |

P388 cells. Cells that were incubated for 24 h in media containing 20 $\mu$M of adenosine or AMP, showed the same level of enzyme activity as untreated cells. These levels of enzyme activity were also found in cells of both lines freshly collected from ascites fluid withdrawn from tumour-bearing $CDF_1$ mice.

In the presence of the detergent Nonident P40 (1%) the enzyme activity increased 3-fold in both cell lines and the relative difference in activity between the lines was maintained.

*Metabolism of arachidonate*

The spontaneous release of $PGE_2$, 6-keto-$PGF_{1\alpha}$ and thromboxane $B_2$ from drug sensitive and resistant cells is shown in Table II. The doxorubicin resistant P388 cells exhibited higher release of all the arachidonate metabolites assayed. The most conspicuous difference observed was in the release of $PGE_2$ (6-fold).

In order to examine a possible correlation between inhibition of growth by doxorubicin and the metabolism of arachidonic acid, cells of both lines were exposed to the drug in concentrations which inhibit the cell growth rate by 50%. Under these conditions, the release of $PGE_2$ from drug-sensitive cells was reduced to 52.2% of that obtained from these cells incubated without doxorubicin. In doxorubicin-resistant cells, the drug inhibited the release of $PGE_2$ by only 18.5%.

Indomethacin, at the highest non-inhibitory concentration ($3 \times 10^{-5}$ M), did not affect the sensitivity of either cell line to doxorubicin.

*Adenosine uptake*

The initial rate of adenosine uptake by doxorubicin-sensitive and -resistant P388 cells, is shown in Table III. This uptake was largely suppressed by *p*-nitrobenzyladenosine, a blocker of adenosine transport. The uptake of adenosine in doxorubicin-resistant cells is considerably higher than that measured in drug-sensitive P388 cells.

Nitrobenzylthioinosine, another blocker of adenosine transport (Lauzon & Paterson, 1977), at the highest non-inhibitory concentration ($3 \times 10^{-6}$ M), did not affect the sensitivity of either cell line to doxorubicin.

### Discussion

The present experiments have shown that the parent P388 cell line, a methylcholanthrene-induced lymphoid neoplasm in DBA/2 mouse (Dawe & Potter, 1957), have measurable 5′nucleotidase activity. This activity is much lower than that measured in normal DBA/2 mouse lymphocytes (Uusitalo & Karnovsky, 1977). The activity of 5′-nucleotidase measured in the doxorubicin-resistant

**Table II** The spontaneous release of arachidonate metabolites from doxorubicin-sensitive and -resistant P388 cells

| | *pg of metabolite $10^6$ cells $3\,h^{-1}$ mean ± s.d. (4 experiments)* | | |
|---|---|---|---|
| | *$PGE_2$* | *6-keto-$PGF_{1\alpha}$* | *thromboxane $B_2$* |
| Doxorubicin-sensitive P388 cells | 46.1 ± 15.8 | 100 ± 10.2 | 2.2 ± 0.3 |
| Doxorubicin-resistant P388 cells | 296.2 ± 22.8 | 200 ± 21.4 | 4.6 ± 0.4 |

**Table III** Adenosine uptake by doxorubicin-sensitive and -resistant P388 cells

| | *pmol adenosine $10^6$ cells $h^{-1}$ mean ± s.d. ($n = 3$)* | |
|---|---|---|
| | *control* | *+ nitrobenzyladenosine* |
| Doxorubicin-sensitive P388 cells | 4.56 ± 0.80 | 1.21 ± 0.04 |
| Doxorubicin-resistant P388 cells | 14.84 ± 2.68 | 1.28 ± 0.36 |

subline, selected by the drug from the parent P388 cell population, was significantly higher than that measured in the parent cell line and was not significantly different from the activity reported for normal DBA/2 mouse lymphocytes.

The activity of this enzyme in either cell line was not affected by prior incubation of the cells in the presence of adenosine or AMP.

The increased activity of 5′-nucleotidase measured in doxorubicin-resistant cells, compared to drug-sensitive cells, may represent an increase in the amount of enzyme protein or an increase in its specific activity as a result of some change in the protein structure or its relation to other constituents of the cell membrane. After solubilization with a detergent, an increase in 5′-nucleotidase activity was measured. However, similar increase in enzyme activity was obtained in both cell lines. A detergent may on one hand increase membrane-bound enzyme activity by increasing its exposure (Solomonson *et al.*, 1976) and on the other hand, may disrupt some enhancing effect of the lipid/protein interaction (Englehard *et al.*, 1976). Therefore this negative result does not support either possibility.

Although this study gives further support for the suggested relationship between the level of activity of 5′-nucleotidase, a membrane-bound enzyme, and the degree of structural order of the cell membrane lipid domain, the explanation for this relationship remains obscure.

The activity of 5′nucleotidase measured in human T- and B-lymphoma cell lines (Carson *et al.*, 1979) was much lower than that reported for the corresponding normal human T- and B-lymphocytes (Thompson *et al.*, 1979; Rowe *et al.*, 1979). It was suggested that the activity level of this enzyme is related to the degree of cell maturity (Poplack *et al.*, 1981). Decreased 5′-nucleotidase activity was reported in most chronic lymphatic leukaemias (Lopez *et al.*, 1973; Marique & Hildebrand, 1973; Quagliata *et al.*, 1974; Kramers *et al.*, 1976), in lymphocytes of immunodeficient patients with hypogammaglobulinemia (Johnson *et al.*, 1977; Edwards *et al.*, 1978; Webster *et al.*, 1978), T cell lymphoblastic leukaemias (Reaman *et al.*, 1979) and B cell acute lymphoblastic leukaemias (Reaman *et al.*, 1981). It was suggested that the activity of this enzyme may be used as a new biological marker of certain diseases (Koya *et al.*, 1981). Our data clearly point toward the possibility that following drug treatment and the emergence of drug-resistant cell populations, the activity of 5′-nucleotidase in the remaining tumour cells may change considerably and an increase in the enzyme activity in the remaining cells may not necessarily indicate a shift toward higher differentiation.

Doxorubicin-resistant P388 cells release more prostaglandin $PGE_2$, thromboxane and prostacyclin than drug-sensitive P388 cells. The differences in arachidonate metabolism may reflect change in the availability of arachidonic acid in these cells as a part of the changes in cell lipid composition (Ramu *et al.*, 1983*b*). However, in preliminary experiments where arachidonic acid was added in excess to the incubation medium, the differences in arachidonate metabolism were maintained. This indicates that the change in arachidonate metabolism is caused by changes in the activity of certain enzymes involved in arachidonate metabolism rather than a difference in the availability of arachidonic acid.

At equicytostatic concentrations of doxorubicin ($ED_{50}$), the release of $PGE_2$ from drug-resistant P388 cells was significantly less influenced by the presence of doxorubicin than its release from drug-sensitive cells. Therefore, we suggest that the inhibition of release of $PGE_2$ by the drug is unrelated to its cytostatic effect. Furthermore as indomethacin did not change the sensitivity of either cell line to doxorubicin, we suggest that the increase in arachidonate metabolism is not the cause of doxorubicin resistance but rather reflects changes in the membrane structure of these cells. Changes in other membrane related activities were found in drug resistant cells as: glycosidase (Bosmann & Kessel, 1970), membrane glycoproteins (Beck *et al.*, 1979), high macromolecular lipid (Taylor *et al.*, 1981) and carrier-mediated uptake of methotrexate (Kessel *et al.*, 1965; Herman *et al.*, 1979). In the present study we have also noted that these cell lines differ in another carrier-mediated uptake system of the cell membrane, namely the uptake of adenosine. The rate of adenosine transport was significantly higher in doxorubicin-resistant cells than in drug-sensitive cells. In both cell lines the uptake could be blocked by a specific adenoside transport blocker, nitrobenzyladenosine. The finding that nitrobenzylthioinosine, another potent inhibitor of nucleosine transport (Lauzon *et al.*, 1977), did not change the sensitivity of either cell line to doxorubucin, suggests that the increase in adenosine uptakes is not the cause of drug resistance, but rather another reflection of the change in membrane structure.

### References

BECK, W.T., MUELLER, T.J. & TANZER, L.R. (1979). Altered surface membrane glycoproteins in vinca alkaloid-resistant human leukemic lymphoblasts. *Cancer Res.*, **39**, 2070.

BELFIELD, A. & GOLDBERG, D.M. (1968). Inhibition of the nucleotidase effect of alkaline phosphatase by β-glycerophosphate. *Nature*, **219**, 73.

BOSMANN, H.B. & KESSEL, D. (1970). Altered glycosidase levels in drug-resistant mouse leukemias. *Mol. Pharmacol.*, **6**, 345.

CARSON, D.A., KAYE, J., MATSUMOTO, S., SEEGMILLER, J.E. & THOMPSON, L. (1979). Biochemical basis for the enhanced toxicity of deoxyribonucleosides toward malignant human T cell lines. *Proc. Natl Acad. Sci.*, **76**, 2430.

DAWE, C.J. & POTTER, M. (1957). Morphologic and biologic progression of a lymphoid neoplasm of the mouse *in vivo* and *in vitro*. *Am. J. Pathol.*, **33**, 603.

EDWARDS, N.L., MAGILAVY, D.B., CASSIDY, J.T. & FOX, I.H. (1978). Lymphocyte ecto-5′nucleotidase deficiency in agammaglobuliemia. *Science*, **201**, 628.

ENGELHARD, V.H., ESKO, J.D., STROM, D.R. & GLASER, M. (1976). Modification of adenylate cyclase activity in LM cells by manipulation of the membrane phospholipid composition *in vivo*. *Proc. Natl Acad. Sci.*, **73**, 4482.

GRANSTROM, E. & KINDHAL, H. (1976). Radioimmunoassay for prostaglandin metabolites. *Adv. Prostaglandin Thromboxane Res.*, **1**, 81.

HERMAN, T.S., CRESS, A.E. & GERNER, E.W. (1979). Collateral sensitivity to methotrexate in cells resistent to adriamycin. *Cancer Res.*, **39**, 1937.

JOHNSON, R.K., OVEJERA, A.A. & GOLDIN, A. (1976). Activity of anthracyclines against an adriamycin (NSC-123127)-resistant subline of P388 leukemia with special emphasis on cinerubin A (NSC-18334). *Cancer Treat. Rep.*, **60**, 99.

JOHNSON, S.M., ASHERTON, G.L., WATTS, R.W.E., NORTH, M.E., ALLSOP, J. & WEBSTER, A.D.B. (1977). Lymphocyte 5′nucleotidase deficiency in primary hypogammaglobulinemia. *Lancet*, **i**, 168.

KESSEL, D., HALL, T.C., ROBERTS, D.W. & WODINSKY, I. (1965). Uptake as a determinant of methotrexate response in mouse leukemias. *Science*, **150**, 752.

KOYA, M., KANOH, T., SAWADA, H., UCHINO, H. & UEDA, K. (1981). Adenosine deaminase and ecto-5′-nucleotidase activities in various leukemias with special reference to blast crisis: Significance of ecto-5′-nucleotidase in lymphoid blast crisis of chronic myeloid leukemia. *Blood*, **58**, 1107.

KRAMERS, M.T.C., CATOVSKY, D., FOA, R., CHERCHI, M. & GALTON, D.A.G. (1976). 5′-nucleotidase activity in leukaemic lymphocytes. *Biomedicine*, **25**, 363.

LAUZON, G.J. & PATERSON, A.R.P. (1977). Binding of the nucleoside transport inhibitor nitrobenzylthioinosine to HeLa cells. *Mol. Pharmacol.*, **13**, 883.

LOPEZ, J., ZUCKER-FRANKLIN, D. & SILBER, R. (1973). Heterogenicity of 5′-nucleotidase activity in lymphocytes in chronic lymphocytic leukaemia. *J. Clin. Invest.*, **52**, 1297.

MARIQUE, D. & HILDEBRAND, J. (1973). Evidence of a 5′-nucleotidase in human leukemic leukocytes. *Clin. Chim. Acta*, **45**, 93.

PETITOU, M., TUY, F., ROSENFELD, C. & 5 others. (1978). Decreased microviscosity of membrane lipids in leukemic cells: Two possible mechanisms. *Proc. Natl Acad. Sci.*, **75**, 2306.

POPLACK, D.G., BLATT, J. & REAMAN, G. (1981). Purine pathway enzyme abnormalities in acute lymphoblastic leukemia. *Cancer Res.*, **41**, 4824.

QUAGLIATA, F., FAIG, D., CONKLYN, M. & SILBER, R. (1974). Studies on the lymphocyte 5′nucleotidase in chronic lymphocytic leukemia, infectious mononucleosis, normal subpopulations and phytohemagglutinin-stimulated cells. *Cancer Res.*, **34**, 3197.

RAMU, A., GLAUBIGER, D., MAGRATH, I.T. & JOSHI, A. (1983*a*). Plasma membrane lipid structural order in doxorubicin-sensitive and resistant P388 cells. *Cancer Res.*, **43**, 5533.

RAMU, A., SHAN, T.-C. & GLAUBIGER, D. (1983*b*). Enhancement of doxorubicin and vinblastine sensitivity in anthracycline-resistant P388 cells. *Cancer Treat. Rep.*, **67**, 895.

RAZ, A., COLLARD, J.G. & INBAR, M. Decrease in 5′-nucleotidase activity in malignant transformed and normal stimulated cells. *Cancer Res.*, **38**, 1258.

REAMAN, G.H., BLATT, J. & POPLACK, D.G. (1981). Lymphoblast purine pathway enzymes in B-cell acute lymphoblastic leukemia. *Blood*, **58**, 330.

REAMAN, G.H., LEVIN, N., MUCHMORE, A., HOLIMAN, B.J. & POPLACK, D.G. (1979). Diminished lymphoblast 5′-nucleotidase activity in acute lymphoblastic leukemia with T-cell characteristics. *N. Engl. J. Med.*, **300**, 1374.

ROWE, M., DE GAST, C.G., PLATTS-MILLS, T.A.E., ASHERTON, G.L., WEBSTER, A.D.B. & JOHNSON, S.M. (1979). 5′-nucleotidase of B and T lymphocytes isolated from human peripheral blood. *Clin. Exp. Immunol.*, **36**, 97.

SOLOMONSON, L.P., LIEPKALNS, V.A. & SPECTOR, A.A. (1976). Changes in $(Na^{+}+K^{+})$-ATPase activity of Ehrlich ascites tumor cells produced by alteration of membrane fatty acid composition. *Biochemistry*, **15**, 892.

TAYLOR, R.F., TEAGUE, L.A. & YESAIR, D.W. (1981). Drug-binding macromolecular lipids from L1210 leukemia tumors. *Cancer Res.*, **41**, 4316.

THOMPSON, L.F., BOSS, G.R., SPIEGLBERG, H.L. & 5 others (1979). Ecto-5′-nucleotidase activity in T and B lymphocytes from normal subjects and patients with congenital X-linked agammaglobulinemia. *J. Immunol.*, **123**, 2475.

UUSITALO, R.J. & KARNOVSKY, M.J. (1977). 5′-nucleotidase in different populations of mouse lymphocytes. *J. Histochem. Cytochem.*, **25**, 97.

WEBSTER, A.D.B., NORTH, M., ALLSOP, J., ASHERTON, G.L. & WATTS, R.W.E. (1978). Purine metabolism in lymphocytes from patients with primary hypogammaglobulinemia. *Clin. Exp. Immunol.*, **31**, 456.